# Supplementary material for: Construction of a recombinant vaccine expressing Nipah virus glycoprotein using the replicative and highly attenuated vaccinia virus strain LC16m8
Source: PLoS Negl Trop Dis. 2023 Dec 15;17(12):e0011851. doi: 10.1371/journal.pntd.0011851 (PMC10756534; doi:10.1371/journal.pntd.0011851)
Supplement: Attachment — Submitted filename: PNTD-D-23-00807 COMMENTS.pdf [file pntd.0011851.s001.pdf]

PLOS Neglected Tropical Diseases

Construction of a recombinant vaccine expressing Nipah virus glycoprotein using the replicative and highly attenuated vaccinia virus strain LC16m8

--Manuscript Draft--

**Manuscript Number:**	PNTD-D-23-00807
**Full Title:**	Construction of a recombinant vaccine expressing Nipah virus glycoprotein using the replicative and highly attenuated vaccinia virus strain LC16m8
**Short Title:**	Generation of a recombinant LC16m8 expressing NiV glycoprotein
**Article Type:**	Research Article
**Keywords:**	nipah virus; vaccinia virus; LC16m8; vaccine; paramyxovirus; poxvirus
**Abstract:**	Nipah virus (NiV) is a highly pathogenic zoonotic virus that causes severe encephalitis and respiratory diseases, and it has a high mortality rate in humans (> 40%). Epidemiological studies on various species of fruit bats, which are natural reservoirs of the virus, have shown that NiV is widely distributed throughout Southeast Asia. Therefore, the development of an effective vaccine against NiV is urgently required. In this study, we generated recombinant vaccinia viruses expressing the NiV G or F protein using the LC16m8 strain and examined their antigenicity or ability to induce immunity. Neutralizing antibodies against NiV were successfully induced in hamsters inoculated with LC16m8 expressing NiV G or F, and their antibody titers were higher than those induced by other vaccinia virus vectors that have been previously reported to prevent lethal NiV infection. The findings indicate that the LC16m8-based vaccine format has superior features as a proliferative vaccine compared with other poxvirus-based vaccines. Moreover, the data collected over the course of antibody elevation during three rounds of vaccination in hamsters provide an important basis for the clinical use of all vaccinia virus-based vaccines against NiV disease.
**Additional Information:**	
**Question**	**Response**
**Government Employee**<br><br>Are you or any of the contributing authors an employee of the United States government?<br><br>Manuscripts authored by one or more US Government employees are not copyrighted, but are licensed under a CC0 Public Domain Dedication, which allows unlimited distribution and reuse of the article for any lawful purpose. This is a legal requirement for US Government employees.<br><br>This will be typeset if the manuscript is accepted for publication.	No - No authors are employees of the U.S. government.
**Financial Disclosure**<br><br>Enter a financial disclosure statement that describes the sources of funding for the	Yes

work included in this submission. Review the submission guidelines for detailed requirements. View published research articles from *PLOS Neglected Tropical Diseases* for specific examples.

This statement is required for submission and **will appear in the published article** if the submission is accepted. Please make sure it is accurate.

Funded studies
Enter a statement with the following details:

- Initials of the authors who received each award
- Grant numbers awarded to each author
- The full name of each funder
- URL of each funder website
- Did the sponsors or funders play any role in the study design, data collection and analysis, decision to publish, or preparation of the manuscript?

Did you receive funding for this work?

Please add funding details.
as follow-up to "**Financial Disclosure**

Enter a financial disclosure statement that describes the sources of funding for the work included in this submission. Review the submission guidelines for detailed requirements. View published research articles from *PLOS Neglected Tropical Diseases* for specific examples.

This statement is required for submission and **will appear in the published article** if the submission is accepted. Please make sure it is accurate.

This study was supported by grants from the Japan Agency for Medical Research and Development (AMED) (JP21fk0108080 <MSH, SW>and 22wm0325002<SW>), Takeda Science Foundation (2017045212<SW> and 2021181927<SW>), Kanae Foundation for the Promotion of Medical Science <SW>, and JSPS KAKENHI (JP22K06016, <SW>).

Powered by Editorial Manager® and ProduXion Manager® from Aries Systems Corporation

**Funded studies**<br>Enter a statement with the following details:<br>• Initials of the authors who received each award<br>• Grant numbers awarded to each author<br>• The full name of each funder<br>• URL of each funder website<br>• Did the sponsors or funders play any role in the study design, data collection and analysis, decision to publish, or preparation of the manuscript?<br><br>Did you receive funding for this work?"	
Please select the country of your main research funder (please select carefully as in some cases this is used in fee calculation).<br>as follow-up to "**Financial Disclosure**<br><br>Enter a financial disclosure statement that describes the sources of funding for the work included in this submission. Review the submission guidelines for detailed requirements. View published research articles from *PLOS Neglected Tropical Diseases* for specific examples.<br><br>This statement is required for submission and **will appear in the published article** if the submission is accepted. Please make sure it is accurate.<br><br>**Funded studies**<br>Enter a statement with the following details:<br>• Initials of the authors who received each award<br>• Grant numbers awarded to each author<br>• The full name of each funder<br>• URL of each funder website<br>• Did the sponsors or funders play any role in the study design, data collection and analysis, decision to publish, or preparation of the manuscript?<br><br>Did you receive funding for this work?"	JAPAN - JP
**Competing Interests**	The authors declare that they have no competing interests.

On behalf of all authors, disclose any competing interests that could be perceived to bias this work.<br><br>This statement will be typeset if the manuscript is accepted for publication.<br><br>Review the instructions link below and PLOS NTDs' competing interests policy to determine what information must be disclosed at submission.	
**Data Availability**<br><br>Provide a **Data Availability Statement** in the box below. This statement should detail where the data used in this submission can be accessed. This statement will be typeset if the manuscript is accepted for publication.<br><br>Before publication, authors are required to make all data underlying their findings fully available, without restriction. Review our PLOS Data Policy page for detailed information on this policy. Instructions for writing your Data Availability statement can be accessed via the Instructions link below.	Data will be made available on request.

Powered by Editorial Manager® and ProduXion Manager® from Aries Systems Corporation

Manuscript
Click here to access/download;Manuscript;Watanabe Shumpei et al LC16m8 NiV PLOS NTD submission ver.docx

Construction of a recombinant vaccine expressing Nipah virus glycoprotein using the replicative and highly attenuated vaccinia virus strain LC16m8

Shumpei Watanabe[1, 2*], Tomoki Yoshikawa[2], Yoshihiro Kaku[3], Takeshi Kurosu[2], Shuetsu Fukushi[2], Satoko Sugimoto[2], Nishisaka Yuki[1], Hikaru Fuji[1], Glenn Marsh[4], Ken Maeda[3], Hideki Ebihara[2], Shigeru Morikawa[1], Masayuki Shimojima[2*], Masayuki Saijo[2, 5]

[1]Department of Microbiology, Faculty of Veterinary Medicine, Okayama University of Science, Japan

[2]Department of Virology I, National Institute of Infectious Diseases, Japan

[3]Division of Veterinary Science, National Institute of Infectious Diseases, Japan

[4]Australian Centre for Disease Preparedness, CSIRO, Australia

[5]Public Health Office, Health and Welfare Bureau, Sapporo Municipal Government, Japan.

Short Title: Generation of a recombinant LC16m8 expressing NiV glycoprotein

Key words: nipah virus, vaccinia virus, LC16m8, vaccine, paramyxovirus, poxvirus

*Address correspondence to Shumpei Watanabe, s-watanabe@ous.ac.jp
or Masayuki Shimojima, shimoji-@niid.go.jp

Word count for Abstract: 129 words

Word count for text: 4660 words

Abstract 186/300 words

Nipah virus (NiV) is a highly pathogenic zoonotic virus that causes severe encephalitis and respiratory diseases, and it has a high mortality rate in humans (> 40%). Epidemiological studies on various species of fruit bats, which are natural reservoirs of the virus, have shown that NiV is widely distributed throughout Southeast Asia. Therefore, the development of an effective vaccine against NiV is urgently required. In this study, we generated recombinant vaccinia viruses expressing the NiV G or F protein using the LC16m8 strain and examined their antigenicity or ability to induce immunity. Neutralizing antibodies against NiV were successfully induced in hamsters inoculated with LC16m8 expressing NiV G or F, and their antibody titers were higher than those induced by other vaccinia virus vectors that have been previously reported to prevent lethal NiV infection. The findings indicate that the LC16m8-based vaccine format has superior features as a proliferative vaccine compared with other poxvirus-based vaccines. Moreover, the data collected over the course of antibody elevation during three rounds of vaccination in hamsters provide an important basis for the clinical use of all vaccinia virus-based vaccines against NiV disease.

Author summary 131/200 words

The Nipah virus (NiV) is an emerging paramyxovirus that is widely observed in Southeast Asia, and it causes fatal respiratory and neurological diseases in humans and animals. The World Health Organization considers NiV disease a priority infectious disease for which an effective vaccine should be developed. LC16m8 is a highly attenuated strain of the vaccinia virus (VV), and more than 100,000 people have been vaccinated with it in Japan without exhibiting severe side effects. Compared to other attenuated VV vaccines,

LC16m8 has a relatively high or modest proliferative potential in mammalian hosts, resulting in the efficient induction of immune responses in humans. In the present study, we successfully generated recombinant LC16m8 expressing surface glycoprotein of NiV and confirmed that these vaccine candidates could efficiently induce neutralizing antibodies against NiV in hamsters.

Introduction

Nipah virus (NiV) is an enveloped virus with a non-segmented negative-strand RNA genome that belongs to the genus *Henipavirus* in the family Paramyxoviridae [1]. NiV has two surface glycoproteins on its envelope, the G and F proteins, which are responsible for receptor binding and membrane fusion, respectively, and considered the major immunogens that induce neutralizing antibodies.

NiV was first discovered in 1998–99 and identified as the causative agent of severe respiratory disease in pigs and encephalitis or respiratory disease in humans (276 recorded cases, 40% mortality) in Malaysia and Singapore [1-3]. Subsequent virus outbreaks have been reported annually in Bangladesh and India. In a few cases, person-to-person transmission through close direct contact has been reported [4, 5]. In Southeast Asia, certain fruit bat species are natural reservoirs of the virus, thus leading to its wide circulation [3, 6-9]. Because of the pandemic potential of the virus based on its specific features, the World Health Organization has prioritized NiV disease in the blueprint of research and development [10]. Currently, therapeutic drugs have not been approved for NiV and vaccines are not available in humans; however, a relatively large number of vaccine candidates have been developed based on recombinant viruses using viral vectors, virus-like particles, and subunit vaccines, and their efficacies have been demonstrated in

experimental animal models (summarized in a review [11]). Recently, NiV vaccines have been developed using mRNA platforms, and one is currently undergoing phase I clinical trials (ClinicalTrials.gov NCT05398796). Each candidate has advantages and disadvantages in terms of vaccine efficacy, safety, and cost-effectiveness. Among them, vaccine vectors based on poxviruses, including a VV (NYVAC strain) and canary poxvirus, have been tested experimentally and shown to induce protection against lethal infection with NiV in hamsters [12, 13].

Until the eradication of smallpox in 1980, multiple strains of live VV were used as smallpox vaccines. However, these first-generation vaccines were associated with serious adverse events [14-17]. Second-generation vaccines, such as ACAM2000, were developed by growing first-generation seeds in cell culture. ACAM2000 is licensed but available only for groups at high risk of variola virus infection. However, these drugs cause relatively frequent and severe side effects [18]. In the late stage of the eradication program, third-generation smallpox vaccines, including those based on the LC16m8 strain and modified VV-Ankara (MVA), were developed as safer candidates [19]. However, the efficacy of these vaccines in preventing smallpox in humans has not been evaluated because eradication has been accomplished using first-generation vaccines. Both LC16m8 and MVA possess a highly attenuated phenotype and immunogenicity that confer protective efficacy against other orthopoxvirus infections [20-24]. In 2019, an MVA-based vaccine, MVA-BN (JYNNEOS) was approved by the Food and Drug Administration for the prevention of smallpox and monkeypox diseases. In the early 1970s, the LC16m8 strain was established from the Lister strain in Japan by multiple passages in primary rabbit kidney cells at low temperature of 30 °C, followed by smaller pock-picking selection on the chorioallantonic membrane of an embryonated egg [22, 25].

One genetic factor responsible for the attenuation of this vaccine is a defect in the expression of the B5R membrane protein [26].

Notably, LC16m8 replicates well in certain types of mammalian cells, such as primary rabbit kidney cells and RK13 cells [22, 25]. In contrast, MVA does not produce progeny viruses in most mammalian cells and only replicates in chicken embryo fibroblast cells, possibly because MVA lost approximately 10% of the genome of the parental Ankara strain [19, 25, 27-29]. Compared with MVA, the growth feature of LC16m8 confers a superior growth capacity in mammalian hosts, including humans, resulting in robust humoral and cellular immune responses, while maintaining high attenuation in humans. In Japan, LC16m8 was approved as a licensed vaccine for smallpox and its safety profiles were evaluated by vaccinating more than 100,000 children, among whom serious adverse events were not reported [22, 30]. Fourth-generation vaccine strains have been established using recombination techniques; for example, strain NYVAC was derived from the Copenhagen strain by deleting 18 non-essential genes and exhibits a similar or poorer growth profile in cell cultures compared to the MVA strain [31] and a highly attenuated phenotype. Third- and fourth-generation smallpox vaccines have also been used as promising vectors to express exogenous viral proteins [32-36]. Some of these vectors, especially those used to establish vaccines against highly pathogenic viruses, have been evaluated as candidates for prophylactic and therapeutic vaccines at the experimental level. Recently, we demonstrated that immunization of a small animal model with recombinant LC16m8s encoding proteins of severe fever with thrombocytopenia syndrome virus (SFTSV) protected against a lethal challenge with SFTSV [36]. Among the aforementioned vector vaccine candidates, the MVA-based vector vaccine MVA-BN-Filo (Johnson & Johnson) was approved for Ebola virus disease

by the European Commission in 2020 and is used as a booster vaccine. In this study, recombinant LC16m8 expressing the NiV surface glycoprotein G or F was constructed, and the induction of neutralizing antibodies against NiV was examined in hamsters inoculated with recombinant LC16m8s. The findings presented here will provide an important basis for the clinical use of all VV-based vaccines against NiV disease.

Materials and methods

Cell lines and viruses

RK13, 293T, and Vero cells were maintained in Dulbecco's modified Eagle's medium (DMEM; Wako, Osaka, Japan) supplemented with 10% fetal bovine serum (FBS; DMEM-10FBS). The LC16m8 strain of VV (accession no. AY678275) [26] were propagated in RK13 cells. The infectious titer of LC16m8 in RK13 cells was determined using a standard plaque-forming unit (PFU) assay [26]. The Malaysian NiV strain Ma-JMR-01-98 was kindly provided by Dr. Kouichi Morita (Institute for Tropical Medicine, Nagasaki University, Nagasaki, Japan). The infectious dose of NiV was determined in Vero cells using a 50% standard tissue culture infectious dose ($TCID_{50}$) assay [37]. Experiments using infectious NiV were conducted in BSL-3 and BSL-4 laboratories under the biosafety regulations of the National Institute of Infectious Diseases (NIID), Tokyo, Japan.

Plasmids

To construct a plasmid encoding either the NiV G or F protein of the Malaysian strain (Accession No. NC-002728) and encoding the EGFP protein driven by early and late VV promoters, a complete gene synthesis service was used (Fasmac, Yokohama, Japan).

Following gene synthesis, which included the promoter sequence and the open reading frame of G or F, each synthesized gene was digested with XhoI or double-digested with SalI and XhoI and then ligated to the plasmid pRecB5R.1 [38], thus producing pRecB5R.1-NiV-G and pRecB5R.1-NiV-F. The plasmid pCAGGS-NiV-F, which encodes the F gene of the NiV Malaysian strain (NC-002728), was previously established [39]. The expression plasmid pCAGGS-NiV-G, which expresses the G protein of the NiV Malaysia strain (NC-002728) with amino acid substitutions (A181G/S553F/E544K), was constructed to replace the Kozak sequence (from accacc to gccacc) based on the plasmid pCAGGS-G [40]. The G gene was amplified by PCR using the primer pair 5′-gagcatgcgccaccATGCCGGCAGAAAACAAGAAAGTTAGATTC-3′ and 5′-CAAGCTAGCTTATGTACATTGCTCTGGTATCTTAACCGCG-3′. The PCR product was digested with SphI and NheI, and inserted into pCAGGS [41].

Generation of recombinant LC16m8 harboring NiV glycoproteins

Recombinant LC16m8s, namely, LC16m8-G and LC16m8-F, were generated from LC16m8 using a homologous recombination technique for foreign gene insertion with excisable selection markers as reported previously [38]. Briefly, 293T cells were transfected with pRecB5R.1-NiV-G or pRecB5R.1-NiV-F using TansIT-LT1 (Mirus Bio, Madison, WI, USA), and the transfected cells were then infected with LC16m8 at a multiplicity of infection (MOI) of 0.05. The cells were cultured in DMEM-10FBS for three days. The culture medium and cells were collected and freeze-thawed thrice to prepare crude intermediate recombinant LC16m8 stocks. RK13 cells were inoculated with crude LC16m8 stock. Subsequently the inoculum was removed and the cells were

overlaid with DMEM containing 2% FBS, 20 μg/ml mycophenolic acid (MPA, Sigma-Aldrich, St Louis, MO, USA), 250 μl/ml xanthine, 15 μg/ml hypoxanthine, and 1% agarose ME (Iwai Chemicals, Tokyo, Japan). After 2–3 days, plaques that were double-positive for mCherry and EGFP fluorescence signals were identified using a fluorescence microscope Eclipse Ts2-FL (Nikon, Tokyo, Japan). The cells constituting the double-positive plaques were collected in 100 μL of DMEM-10FBS. Plaque purification with the selected drugs (MPA, xanthine, and hypoxanthine) was repeated twice and the plaque was stored as an intermediate recombinant virus while retaining the selection cassette (mCherry and XGPRT). The resultant virus was further propagated in RK13 cells without the addition of the above-mentioned selection drugs to remove the selection cassette via spontaneous self-excision. After three freeze-thaw cycles, the supernatant including infectious recombinant LC16m8 was harvested by centrifugation at 2,000×*g* and 4 °C for 10 min, and aliquots were stored as working stocks.

Indirect immunofluorescence assay (IFA) to confirm expression of NiV glycoproteins

RK13 cells cultured in 6-well plates were infected with LC16m8-G or LC16m8-F at an MOI of 0.01. At 40 h postinfection (hpi), the cells were washed with PBS three times and fixed in PBS containing 2% formaldehyde. The cells were then blocked with 1% normal donkey serum (NDS) in PBS (PBS-1%NDS) overnight at 4 °C. After removal of the blocking buffer, the cells were incubated with polyclonal rabbit serum containing anti-NiV G antibodies or anti-NiV F antibodies in PBS-1%NDS for 1 h at room temperature [39]. After three washes with PBS, the cells were incubated with Alexa Fluor 488-conjugated goat anti-rabbit IgG (H+L) (Invitrogen, Carlsbad, CA, USA) in PBS-1%NDS

for 1 h at room temperature. After washing with PBS, the stained cells were observed under a fluorescence microscope (Eclipse Ts2-FL).

Animal experiments

Hamsters were used to evaluate the immunogenicity of the LC16m8-based NiV vaccine because a hamster infection model was previously established for the NiV challenge test [42]. Female 4–5-week-old Syrian golden hamsters (SLC-Japan, Shizuoka, Japan) were used at the time of the primary injection of LC16m8 or recombinant LC16m8s in all animal experiments. First, for the evaluation of antibody induction by the recombinant viruses, 3 groups of hamsters (Group G-single-site, F-single-site, and G/F-single-site) were inoculated once or twice with the virus solution of 100 μl of DMEM-10FBS, which contained a total of 5×10^6 PFU of the recombinant viruses (LC16m8-G, LC16m8-F, or both viruses containing equal PFU) intramuscularly at 2-week intervals. Similarly, three groups of hamsters harboring pre-existing immunity against VV were inoculated once or twice at 2-week intervals with the viral solution containing 5×10^6 PFU of each recombinant virus or the vehicle control containing DMEM-10FBS only (Group VV-G-single-site, VV-G/F-single-site, and VV-mock-single-site). Similarly, three groups of hamsters harboring pre-existing immunity against VV were inoculated once or twice at 2-week intervals with the viral solution containing 5×10^6 PFU of each recombinant virus or the vehicle control containing DMEM-10FBS only (Group VV-G-single-site, VV-G/F-single-site, and VV-mock-single-site). Four weeks before the prime injections of recombinant viruses, hamsters were intramuscularly inoculated with 100 μl of DMEM-10FBS containing 1×10^6 PFU of LC16m8 at the right hindlimb to endow preexisting immunity. All injections were performed under anesthesia with combination drugs (0.3

mg/kg of medetomidine, 4.0 mg/kg of midazolam, and 5.0 mg/kg of butorphanol). Two weeks after the final dose of recombinant viruses, hamsters were euthanized using a combination of anesthetics, and serum was collected.

For the evaluation of antibody induction during a series of three-time immunizations with the NiV glycoprotein-expressing LC16m8s in VV-preimmunized hamsters, at first, hamsters were injected intramuscularly with 100 µl of DMEM containing 1×10^6 PFU of LC16m8 in the right hindlimb for preimmunization. Four weeks after inoculation, VV-preimmunized hamsters in Group 1 (VV-G-single-site) and Group 2 (VV-F-single-site) were injected with 200 µl of DMED-10FBS containing 2×10^6 PFU of LC16m8-G and that of the LC16m8-F, respectively. The VV-preimmunized hamsters in Group 3 (VV-G-F-separate-sites) were injected with 100 µl of DMEM containing 1×10^6 PFU of LC16m8-G and LC16m8-F) at the right and left hindlimbs, respectively. These injections were repeated 1–3 times at a 2-week interval. Two weeks after the last injection of the designated recombinant virus, serum samples were collected as described above. All animal experiments were reviewed and approved by the Animal Experiments Committee of Okayama University of Science and were carried out in accordance with the Regulations for Animal Experiments of Okayama University of Science (#2019-75, 2020-57, 2021-72, 2023-1).

Measurement of antibody titers in hamster serum

Serial dilutions of hamster serum were prepared and virus neutralization tests were performed (final volume: 50 µL/well). Each sample was added at a ratio of 1:2 to the dilution media (DMEM-10FBS), and quadruplicate two-fold serial dilutions were performed. An equal volume of dilution medium containing 100 $TCID_{50}$ of NiV was

added to each well and incubated 1 h at 37 °C. Virus-serum mixtures were added to confluent Vero cells seeded in a 96-well plate. The 50% neutralization dose (ND_{50}) for 50 μl of hamster serum was calculated according to the Behrens-Kärber method [43]. Neutralization titers of each serum sample were also determined using a previously established VSV-NiV-SEAP assay [44].

To determine the IFA titers of antibodies induced in hamster serum, BHK cells cultured in a 96-well plate were transfected with 0.1 μg of pCAGGS-NiV-G or pCAGGS-NiV-F using TansIT-LT1 (Mirus Bio). At 24-36 h posttransfection, the cells were washed with PBS three times, fixed with PBS containing 2% formaldehyde, and then blocked by PBS-1%NDS overnight at 4 °C. The cells were then incubated with polyclonal rabbit serum containing anti-NiV G antibodies or anti-NiV F antibodies in PBS-1%NDS for 1 h at room temperature [39]. After washing with PBS 3 times, the cells were incubated with Alexa Fluor 488-conjugated goat anti-hamster IgG (H+L) (Invitrogen) in PBS-1%NDS for 1 h at room temperature. After washing three times with PBS, the stained cells were observed under a fluorescence microscope (BZ-X810, Keyence, Osaka, Japan). The IFA titer was defined as the reciprocal of the highest serum dilution at which the fluorescence signal was detected.

Statistical analysis

Data were analyzed, and final graphs were prepared using GraphPad Prism (version 9.3.1) software. One-tailed Student's *t*-tests (unpaired test with Welch's correction) were used to compare means between the two groups. A P-value < 0.05 was considered significant. The number of animals in each group and specific details of the statistical tests are indicated in the figure legends.

Results

Generation of recombinant LC16m8s expressing NiV surface glycoproteins

We previously reported a system for constructing a recombinant LC16m8 coding foreign genes based on a homologous recombination technique, in which the target sequence was inserted into the region of the B5R gene of the LC16m8 genome [38]. During the construction process, the recombinant clone harboring the gene of interest was selected by the expression of the mCherry gene under the influence of selection drugs (MPA, xanthine, and hypoxanthine), and the cassette of selection markers (mCherry and XGPRT) was self-excised in the culture without the selection drugs to yield the final recombinant virus (Fig 1). Using this method, the complete gene coding sequence of G of the NiV Malaysia strain or NiV-F was inserted into the LC16m8 genome. Subsequently, intermediate recombinant viruses (with the selection cassette) were independently subjected to three cycles of plaque purification using the selected drugs. After plaque purification, each of the resulting viruses was grown once in culture without the selected drugs, and each virus was stored as working stock. In poxviruses, gene loss can occur through insertions, deletions, or point mutations [45]. To monitor the occurrence of the deletion of target foreign genes or contamination with the original LC16m8 during successive cultures of the virus stock, the gene coding sequence of EGFP was inserted into the LC16m8 genomes (Fig 1). By checking the EGFP fluorescence signal, we confirmed that cytopathic effects (CPEs) without EGFP expression were not detected or were limited, suggesting that the occurrence of gene deletion and contamination of the original virus (LC16m8) in the working virus stock was negligible or insignificant.

The initial working stocks of the recombinant LC16m8s, namely LC16m8 carrying NiV G (LC16m8-G) and LC16m8 carrying NiV F (LC16m8-F), were confirmed to express the NiV G and F proteins in the infected cells by IFA using antibodies against G and F, respectively (Fig 2A and B). Moreover, the formation of multinucleated giant cells was notably observed in RK13 cells transfected with the plasmid encoding the NiV-G or NiV-F genes under the VV early and late promoters (pRecB5R.1-NiV-G or pRecB5R.1-NiV-F) after infection with recombinant LC16m8-F or LC16m8-G, respectively (Fig 2C). In addition, syncytium formation induced by LC16m8 was not significant in RK13 cells. This suggests that the NiV glycoprotein expressed in cultured cells infected with LC16m8-G or LC16m8-F retains its original function of inducing membrane fusion. Although we attempted to establish recombinant LC16m8 expressing both the NiV G and F proteins simultaneously, no virus clones were obtained after the initial homologous recombination step (Fig 1).

Immunization of hamsters with LC16m8-G or LC16m8-F alone or in combination at one site

Four weeks after the inoculation of hamsters with the VV strain LC16m8 (1×10^6 plaque forming unit (PFU)/hamster), three groups of hamsters (VV-G-single-site, VV-G/F-single-site, and VV-mock-single-site) were intramuscularly inoculated with 5×10^6 PFU of LC16m8-G, a mixture of LC16m8-G and LC16m8-F (2.5×10^6 PFU each), and the DMED-10FBS growth medium, respectively (Fig 3A). Two weeks post-inoculation, serum samples were collected from half of the hamsters in each group and inoculation with the designated recombinant viruses or negative controls was repeated for the remaining hamsters in each group. Two weeks after the second inoculation, serum

samples were collected and tested via neutralizing assay. Using infectious NiV or the vesicular stomatitis virus (VSV) pseudovirus bearing the NiV G and F proteins (VSV-NiV-SEAP) [44], the titer of the neutralizing antibody was determined (SNT or pVSV-SNT) (Fig 3B and C).

Two immunizations of the VV-pre-immunized hamsters with LC16m8-G barely induced neutralizing antibodies to NiV (Group VV-G-single-site) (Fig 3B), whereas pVSV-SNT was detected by the surrogate assay (Fig 3C). In addition, neutralizing antibodies were not detected in hamsters inoculated with the vehicle controls (Group VV-mock-single-site) (Fig 3B), although a non-specific reaction was detected in hamsters inoculated with VSV-NiV-SEAP (<32) (Fig 3C). These results suggest that preexisting immunity against VV greatly impairs the antibody response against NiV. The induction of SNT and pVSV-SNT by the mixture of LC16m8-G and LC16m8-F in the VV-G/F-single-site group was slightly higher than that by LC16m8-G in the VV-G-single-site group, although no statistically significant differences were detected (Fig 3B and C).

To evaluate the immunogenicity of LC16m8-F and LC16m8-G more precisely, hamsters were inoculated with LC16m8-G (Group G-single-site), LC16m8-F (Group F-single-site), or a mixture of LC16m8-G and LC16m8-F (Group G/F-single-site) once or twice at 2-week intervals without prior LC16m8 inoculation (Fig 3D). Two weeks after the final inoculation, serum samples were collected, and SNT and pVSV-SNT were determined (Fig 3E and F). All SNTs detected after booster immunization shown in Fig 3E were higher (>4) than those detected in Fig 3B. However, SNTs were still below the limit of detection (<2) in one or two serum samples collected after booster immunization in each group, indicating that neither LC16m8-G nor LC16m8-F efficiently neutralized NiV. The induction of both SNTs and pVSV-SNTs by LC16m8-F appeared to be slightly higher

than that by LC16m8-G, although the difference was not significant (Fig 3E and F).

Inoculation of VV-preimmunized hamsters with LC16m8-G or LC16m8-F alone or both viruses at two separate sites

Since two cycles of immunization with LC16m8-G or LC16m8-F only induced low levels of antibody reactions in hamsters, especially with prior immunization with the parental VV (Fig 3B and C), we tested three immunization cycles with the recombinant virus in hamsters preimmunized with LC16m8 (Fig 4A). The results in Figure 3B and C indicated that the immune response induced by the mixture of LC16m8-G and LC16m8-F would neutralize NiV infectivity more efficiently because the antibodies induced by both proteins might function synergistically. However, synergistic effects were not observed or confirmed in the subsequent experiments (Fig 3E and F). Here, we hypothesized that syncytium formation caused by NiV-G and NiV-F proteins expressed in cells following infection with LC16m8-G and LC16m8-F would detrimentally affect the induction of antibody reactions. Thus, we tested another inoculation method in hamsters, which included simultaneous inoculation with LC16m8-G and LC16m8-F at different sites (Fig 4A). Four weeks after the preceding immunization of 27 hamsters with LC16m8 (1×10^6 PFU/hamster), eight and nine of the 27 hamsters were inoculated with 2×10^6 PFU of LC16m8-G and LC16m8-F intramuscularly in the right hindlimb, respectively (Group 1: VV-G-single-site, and Group 2: VV-F-single-site) (Fig 4A). The remaining 10 hamsters were inoculated with 1×10^6 PFU of LC16m8-G and the same titer of LC16m8-F separately but simultaneously in the right and left hind limbs (Group 3: VV-G-F-separate-sites). One-third of the hamsters in each group was further inoculated once or twice in the same manner as the initial inoculation with the same recombinant LC16m8s. Two weeks

after the last inoculation, serum samples were collected at the designated time points and subjected to neutralization tests (Fig 4A). Although three immunization cycles with LC16m8-G induced strong antibody reactions in two hamsters in Group 1 (>16), one remaining hamster did not respond to the inoculations (Fig 4B). In contrast, neutralizing antibodies were induced in all three hamsters after three shots with LC16m8-F. However, two injections of LC16m8-F did not induce a reaction in any of the three hamsters in Group 2. In contrast, double or triple inoculation with the simultaneous inoculation method using LC16m8-G and LC16m8-F at different sites efficiently induced neutralizing antibodies against NiV and produced high titers in all hamsters in Group 3 (Fig 4B). These results indicate that some hamsters immunized with LC16m8-G or LC16m8-F once or twice did not produce any neutralizing antibodies in their sera. Here, we examined whether antibodies (other than the neutralizing antibody) were induced in these hamsters' sera (Fig 4C). BHK cells transiently expressing the NiV G or F protein were reacted with dilutions of hamster serum, and antibodies against both proteins were detected using IFA. Although the IFA antibody against the G protein was not detected in hamsters that received a single immunization with LC16m8-G, the antibodies (256 and 512) were detected in hamsters with two and three cycles of the immunization with LC16m8-G (Fig 4C).

Discussion

In this study, we succeeded in generating LC16m8-G and LC16m8-F and confirmed the expression of the G or F proteins in cells infected with either of the recombinant LC16m8s (Fig 1 and 2). Moreover, neutralizing antibodies against NiV were induced in all hamsters inoculated three times with these recombinant viruses (Fig 4B). In parallel with the

increase in neutralizing antibodies during the three immunizations, an increase in IFA antibody titers was observed (Fig 4C). However, the levels of neutralizing antibodies induced by one or two inoculations of each recombinant LC16m8 were relatively low or below the detection limit, although IFA antibodies were detected in all hamsters except for one hamster inoculated once with LC16m8-G (Fig 4C). The levels of neutralizing antibodies induced by LC16m8-based vaccine candidates in the present study were higher than or similar to those induced in hamsters inoculated with the NYVAC-based vaccine expressing the NiV glycoprotein in a previous report [12]. Two cycles of inoculation with NiV G-expressing NYVAC (NYVAC-NiV-G) or NiV F-expressing NYVAC (NYVAC-NiV-F) have been shown to induce complete protection against lethal NiV infection in hamsters [12]. This suggests that the recombinant LC16m8 developed in this study has the potential to protect hamsters from lethal NiV infection after only two cycles of immunization, similar to the NYVAC-based vaccine. When we increased the number of immunization cycles to three, NTs were elevated (more than 16-fold) after the last cycle. As SNTs were determined or measured with complete inhibition of the cytopathic effect in this study, hamsters inoculated with three immunizations of recombinant LC16m8s were expected to survive after lethal infection with NiV, even if predicted or anticipated only in terms of the humoral immune response. However, further challenge studies with NiV are necessary to confirm the efficacy of recombinant LC16m8s as an NiV vaccine.

Originally, LC16m8 grew less efficiently in hamsters and mice than in humans [25]. LC16m8 inoculation induces local skin reactions and erythema in humans [30], whereas it does not induce any clinical symptoms, including skin reactions, in hamsters and mice in the current and previous studies [36]. This suggests that hamster and mouse models underestimate the potency of recombinant LC16m8 in inducing neutralizing antibodies

because of the limited growth features of the virus vector in these animals.

MVA lost approximately 10% of the genome of the parental Ankara strain, especially at both end regions of the genome [46, 47]. As a result, MVA does not grow in most cell cultures, except in chicken embryo fibroblasts and BHK-21 cells [27-29, 48]. Furthermore, the defective regions contain several genes associated with evasion of the innate immune system, thus leading to the enhancement of antigen presentation and immunogenicity and efficient induction of acquired immunity. These characteristics of MVA may lead to improved vaccine safety and efficacy. MVA is the most widely used platform for VV-based vaccines in clinical practice. In the NYVAC strain, 18 open reading frames from the parental Copenhagen strain are deleted, and these deleted genes are involved in pathogenicity, virulence, and host range [31]. Compared to the MVA strain, the growth profile of NYVAC in cell culture was comparable or inferior, thus showing similar proliferative features [48]. In contrast, LC16m8 maintains proliferative activity in some cultured cells, including primary rabbit kidney cells, and has higher proliferative potential than MVA and NYVAC [24]. Compared to the parental strain, LC16m8 lacks the B5R protein (one of the major neutralizing antigens) because of a frameshift mutation with a single gene deletion [26]. Although gene defects are thought to be one of the main causes of attenuation in the LC16m8 strain, the mechanism of attenuation raises concerns regarding reversion to the parental strain through reversion mutations. Nevertheless, clinical trials conducted in Japan have demonstrated that its safety is maintained [22, 30]. Another concern about the decreased induction of neutralizing antibodies is associated with the loss of the neutralizing antigen, although LC16m8 has been reported to maintain the ability to induce humoral and cellular immunity [49]. The occurrence of reversion at a very low rates may enhance the induction of humoral responses without enhancing

virulence. Notably, in studies reporting the efficacy of LC16m8 or recombinant LC16m8, the input of viral doses was usually 10-100 times lower than that of MVA or NYVAC [36, 49, 50]. Taken together, LC16m8 is a promising viral vector vaccine format that can induce a stronger humoral immune response based on its relatively high or modest proliferative potential compared to other attenuated VV-based vaccines. In addition, an attenuated canary poxvirus expressing the glycoprotein of Hendra virus (a closely related species of NiV) has been reported [13]. Canary poxviruses have low proliferative potential in host animals, except for avian species. All these facts suggest that the LC16m8-based vaccine format has superior features as a proliferative vaccine compared with other poxvirus-based vaccines.

In the present study, antibody titers induced by LC16m8-G tended to be lower than those induced by LC16m8-F, although no significant differences were detected. This trend is consistent with previous reports on NYVAC-based vaccines [12]. Interestingly, although LC16m8-G may be less effective than LC16m8-F in inducing neutralizing antibodies, the most robust induction of neutralizing antibodies was observed in hamsters that were inoculated simultaneously with LC16m8-G and LC16m8-F, but not in hamsters inoculated with LC16m8-F alone (Fig 4B). Notably, LC16m8-G, F, which carries both the G and F genes, was not rescued in this study (Fig 1). These facts suggest that the growth of LC16m8 is restricted to the local site where G and F are expressed and syncytia formation is induced. However, further studies are required to verify this possibility.

Author Contributions

Conceptualization: Shumpei Watanabe, Tomoki Yoshikawa, Yoshihiro Kaku, Takeshi Kurosu, Hikaru Fuji, Shigeru Morikawa, Masayuki Shimojima, Masayuki Saijo.

Formal analysis: Shumpei Watanabe, Yoshihiro Kaku.

Funding acquisition: Shumpei Watanabe, Masayuki Shimojima.

Investigation: Shumpei Watanabe, Tomoki Yoshikawa, Yoshihiro Kaku, Takeshi Kurosu, Shuetsu Fukushi, Satoko Sugimoto, Nishisaka Yuki, Masayuki Shimojima

Project administration: Shumpei Watanabe, Hideki Ebihara, Ken Maeda, Masayuki Shimojima, Masayuki Saijo.

Resources: Tomoki Yoshikawa, Glenn Marsh

Supervision: Masayuki Shimojima, Masayuki Saijo.

Visualization: Shumpei Watanabe, Yoshihiro Kaku.

Writing – original draft: Shumpei Watanabe, Masayuki Saijo.

Writing – review & editing: Shumpei Watanabe, Tomoki Yoshikawa, Yoshihiro Kaku, Takeshi Kurosu, Shuetsu Fukushi, Satoko Sugimoto, Nishisaka Yuki, Hikaru Fuji, Glenn Marsh, Ken Maeda, Hideki Ebihara, Shigeru Morikawa, Masayuki Shimojima, Masayuki Saijo

Data availability

Data will be made available on request.

Declaration of Competing Interest

The authors declare that they have no competing interests.

Acknowledgments

We thank Ms. Momoko Ogata of the National Institute of Infectious Diseases of Japan for her assistance in this study. We are grateful to Dr. Kouichi Morita of Nagasaki

University for providing Nipah virus. We would like to thank Editage (www.editage.com) for the English language editing. This study was supported by grants from the Japan Agency for Medical Research and Development (AMED) (JP21fk0108080 and 22wm0325002), Takeda Science Foundation, Kanae Foundation for the Promotion of Medical Science, and JSPS KAKENHI (JP22K06016).

References

1. Amarasinghe GK, Ayllon MA, Bao Y, Basler CF, Bavari S, Blasdell KR, et al. Taxonomy of the order Mononegavirales: update 2019. Arch Virol. 2019;164(7):1967-80. Epub 2019/05/16. doi: 10.1007/s00705-019-04247-4. PubMed PMID: 31089958; PubMed Central PMCID: PMCPMC6641539.

2. Ang BSP, Lim TCC, Wang L. Nipah Virus Infection. J Clin Microbiol. 2018;56(6). Epub 2018/04/13. doi: 10.1128/JCM.01875-17. PubMed PMID: 29643201; PubMed Central PMCID: PMCPMC5971524.

3. Luby SP, Gurley ES. Epidemiology of henipavirus disease in humans. Curr Top Microbiol Immunol. 2012;359:25-40. Epub 2012/07/04. doi: 10.1007/82_2012_207. PubMed PMID: 22752412.

4. Gurley ES, Montgomery JM, Hossain MJ, Bell M, Azad AK, Islam MR, et al. Person-to-person transmission of Nipah virus in a Bangladeshi community. Emerging infectious diseases. 2007;13(7):1031-7. Epub 2008/01/25. doi: 10.3201/eid1307.061128. PubMed PMID: 18214175; PubMed Central PMCID: PMCPMC2878219.

5. Luby SP, Gurley ES, Hossain MJ. Transmission of human infection with Nipah virus. Clinical infectious diseases : an official publication of the Infectious Diseases Society of America. 2009;49(11):1743-8. Epub 2009/11/06. doi: 10.1086/647951.

PubMed PMID: 19886791; PubMed Central PMCID: PMCPMC2784122.

6. Hasebe F, Thuy NT, Inoue S, Yu F, Kaku Y, Watanabe S, et al. Serologic evidence of nipah virus infection in bats, Vietnam. Emerging infectious diseases. 2012;18(3):536-7. Epub 2012/03/02. doi: 10.3201/eid1803.111121. PubMed PMID: 22377109; PubMed Central PMCID: PMCPMC3309638.

7. Reynes JM, Counor D, Ong S, Faure C, Seng V, Molia S, et al. Nipah virus in Lyle's flying foxes, Cambodia. Emerging infectious diseases. 2005;11(7):1042-7. Epub 2005/07/19. doi: 10.3201/eid1107.041350. PubMed PMID: 16022778; PubMed Central PMCID: PMCPMC3371782.

8. Sendow I, Field HE, Curran J, Darminto, Morrissy C, Meehan G, et al. Henipavirus in Pteropus vampyrus bats, Indonesia. Emerging infectious diseases. 2006;12(4):711-2. Epub 2006/05/24. doi: 10.3201/eid1204.051181. PubMed PMID: 16715584; PubMed Central PMCID: PMCPMC3294701.

9. Wacharapluesadee S, Samseeneam P, Phermpool M, Kaewpom T, Rodpan A, Maneeorn P, et al. Molecular characterization of Nipah virus from Pteropus hypomelanus in Southern Thailand. Virology journal. 2016;13:53. Epub 2016/03/27. doi: 10.1186/s12985-016-0510-x. PubMed PMID: 27016237; PubMed Central PMCID: PMCPMC4807597.

10. Mehand MS, Al-Shorbaji F, Millett P, Murgue B. The WHO R&D Blueprint: 2018 review of emerging infectious diseases requiring urgent research and development efforts. Antiviral research. 2018;159:63-7. Epub 2018/09/28. doi: 10.1016/j.antiviral.2018.09.009. PubMed PMID: 30261226; PubMed Central PMCID: PMCPMC7113760.

11. Satterfield BA, Dawes BE, Milligan GN. Status of vaccine research and

development of vaccines for Nipah virus. Vaccine. 2016;34(26):2971-5. Epub 2016/03/15. doi: 10.1016/j.vaccine.2015.12.075. PubMed PMID: 26973068.

12. Guillaume V, Contamin H, Loth P, Georges-Courbot MC, Lefeuvre A, Marianneau P, et al. Nipah virus: vaccination and passive protection studies in a hamster model. Journal of virology. 2004;78(2):834-40. Epub 2003/12/25. doi: 10.1128/jvi.78.2.834-840.2004. PubMed PMID: 14694115; PubMed Central PMCID: PMCPMC368848.

13. Guillaume-Vasselin V, Lemaitre L, Dhondt KP, Tedeschi L, Poulard A, Charreyre C, et al. Protection from Hendra virus infection with Canarypox recombinant vaccine. NPJ Vaccines. 2016;1:16003. Epub 2016/07/28. doi: 10.1038/npjvaccines.2016.3. PubMed PMID: 29263849; PubMed Central PMCID: PMCPMC5707888.

14. Casey CG, Iskander JK, Roper MH, Mast EE, Wen XJ, Torok TJ, et al. Adverse events associated with smallpox vaccination in the United States, January-October 2003. JAMA. 2005;294(21):2734-43. Epub 2005/12/08. doi: 10.1001/jama.294.21.2734. PubMed PMID: 16333009.

15. Halsell JS, Riddle JR, Atwood JE, Gardner P, Shope R, Poland GA, et al. Myopericarditis following smallpox vaccination among vaccinia-naive US military personnel. JAMA. 2003;289(24):3283-9. Epub 2003/06/26. doi: 10.1001/jama.289.24.3283. PubMed PMID: 12824210.

16. Neff J, Modlin J, Birkhead GS, Poland G, Robertson RM, Sepkowitz K, et al. Monitoring the safety of a smallpox vaccination program in the United States: report of the joint Smallpox Vaccine Safety Working Group of the advisory committee on immunization practices and the Armed Forces Epidemiological Board. Clinical infectious diseases : an official publication of the Infectious Diseases Society of America. 2008;46

Suppl 3:S258-70. Epub 2008/03/20. doi: 10.1086/524749. PubMed PMID: 18284367.

17. Sejvar JJ, Labutta RJ, Chapman LE, Grabenstein JD, Iskander J, Lane JM. Neurologic adverse events associated with smallpox vaccination in the United States, 2002-2004. JAMA. 2005;294(21):2744-50. Epub 2005/12/08. doi: 10.1001/jama.294.21.2744. PubMed PMID: 16333010.

18. Petersen BW, Harms TJ, Reynolds MG, Harrison LH. Use of Vaccinia Virus Smallpox Vaccine in Laboratory and Health Care Personnel at Risk for Occupational Exposure to Orthopoxviruses - Recommendations of the Advisory Committee on Immunization Practices (ACIP), 2015. MMWR Morb Mortal Wkly Rep. 2016;65(10):257-62. Epub 2016/03/18. doi: 10.15585/mmwr.mm6510a2. PubMed PMID: 26985679.

19. Sanchez-Sampedro L, Perdiguero B, Mejias-Perez E, Garcia-Arriaza J, Di Pilato M, Esteban M. The evolution of poxvirus vaccines. Viruses. 2015;7(4):1726-803. Epub 2015/04/09. doi: 10.3390/v7041726. PubMed PMID: 25853483; PubMed Central PMCID: PMCPMC4411676.

20. Belyakov IM, Earl P, Dzutsev A, Kuznetsov VA, Lemon M, Wyatt LS, et al. Shared modes of protection against poxvirus infection by attenuated and conventional smallpox vaccine viruses. Proceedings of the National Academy of Sciences of the United States of America. 2003;100(16):9458-63. Epub 2003/07/19. doi: 10.1073/pnas.1233578100. PubMed PMID: 12869693; PubMed Central PMCID: PMCPMC170940.

21. Earl PL, Americo JL, Wyatt LS, Eller LA, Whitbeck JC, Cohen GH, et al. Immunogenicity of a highly attenuated MVA smallpox vaccine and protection against monkeypox. Nature. 2004;428(6979):182-5. Epub 2004/03/12. doi: 10.1038/nature02331.

PubMed PMID: 15014500.

22. Kennedy JS, Gurwith M, Dekker CL, Frey SE, Edwards KM, Kenner J, et al. Safety and immunogenicity of LC16m8, an attenuated smallpox vaccine in vaccinia-naive adults. J Infect Dis. 2011;204(9):1395-402. Epub 2011/09/17. doi: 10.1093/infdis/jir527. PubMed PMID: 21921208; PubMed Central PMCID: PMCPMC3218648.

23. Saijo M, Ami Y, Suzaki Y, Nagata N, Iwata N, Hasegawa H, et al. LC16m8, a highly attenuated vaccinia virus vaccine lacking expression of the membrane protein B5R, protects monkeys from monkeypox. Journal of virology. 2006;80(11):5179-88. Epub 2006/05/16. doi: 10.1128/JVI.02642-05. PubMed PMID: 16698998; PubMed Central PMCID: PMCPMC1472157.

24. Wyatt LS, Earl PL, Eller LA, Moss B. Highly attenuated smallpox vaccine protects mice with and without immune deficiencies against pathogenic vaccinia virus challenge. Proceedings of the National Academy of Sciences of the United States of America. 2004;101(13):4590-5. Epub 2004/04/09. doi: 10.1073/pnas.0401165101. PubMed PMID: 15070762; PubMed Central PMCID: PMCPMC384791.

25. Kenner J, Cameron F, Empig C, Jobes DV, Gurwith M. LC16m8: an attenuated smallpox vaccine. Vaccine. 2006;24(47-48):7009-22. Epub 2006/10/21. doi: 10.1016/j.vaccine.2006.03.087. PubMed PMID: 17052815; PubMed Central PMCID: PMCPMC7115618.

26. Morikawa S, Sakiyama T, Hasegawa H, Saijo M, Maeda A, Kurane I, et al. An attenuated LC16m8 smallpox vaccine: analysis of full-genome sequence and induction of immune protection. Journal of virology. 2005;79(18):11873-91. Epub 2005/09/06. doi: 10.1128/JVI.79.18.11873-11891.2005. PubMed PMID: 16140764; PubMed Central

PMCID: PMCPMC1212643.
27. Blanchard TJ, Alcami A, Andrea P, Smith GL. Modified vaccinia virus Ankara undergoes limited replication in human cells and lacks several immunomodulatory proteins: implications for use as a human vaccine. J Gen Virol. 1998;79 (Pt 5):1159-67. Epub 1998/05/29. doi: 10.1099/0022-1317-79-5-1159. PubMed PMID: 9603331.
28. Carroll MW, Moss B. Host range and cytopathogenicity of the highly attenuated MVA strain of vaccinia virus: propagation and generation of recombinant viruses in a nonhuman mammalian cell line. Virology. 1997;238(2):198-211. Epub 1997/12/24. doi: 10.1006/viro.1997.8845. PubMed PMID: 9400593.
29. Drexler I, Heller K, Wahren B, Erfle V, Sutter G. Highly attenuated modified vaccinia virus Ankara replicates in baby hamster kidney cells, a potential host for virus propagation, but not in various human transformed and primary cells. J Gen Virol. 1998;79 (Pt 2):347-52. Epub 1998/02/24. doi: 10.1099/0022-1317-79-2-347. PubMed PMID: 9472619.
30. Saito T, Fujii T, Kanatani Y, Saijo M, Morikawa S, Yokote H, et al. Clinical and immunological response to attenuated tissue-cultured smallpox vaccine LC16m8. JAMA. 2009;301(10):1025-33. Epub 2009/03/13. doi: 10.1001/jama.2009.289. PubMed PMID: 19278946.
31. Tartaglia J, Perkus ME, Taylor J, Norton EK, Audonnet JC, Cox WI, et al. NYVAC: a highly attenuated strain of vaccinia virus. Virology. 1992;188(1):217-32. Epub 1992/05/01. doi: 10.1016/0042-6822(92)90752-b. PubMed PMID: 1566575.
32. Boudewijns R, Perez P, Lazaro-Frias A, Van Looveren D, Vercruysse T, Thibaut HJ, et al. MVA-CoV2-S Vaccine Candidate Neutralizes Distinct Variants of Concern and Protects Against SARS-CoV-2 Infection in Hamsters. Front Immunol. 2022;13:845969.

Epub 2022/04/05. doi: 10.3389/fimmu.2022.845969. PubMed PMID: 35371064; PubMed Central PMCID: PMCPMC8966703.

33. Dowall SD, Graham VA, Rayner E, Hunter L, Watson R, Taylor I, et al. Protective effects of a Modified Vaccinia Ankara-based vaccine candidate against Crimean-Congo Haemorrhagic Fever virus require both cellular and humoral responses. PLoS One. 2016;11(6):e0156637. Epub 2016/06/09. doi: 10.1371/journal.pone.0156637. PubMed PMID: 27272940; PubMed Central PMCID: PMCPMC4896484.

34. Kitabatake M, Inoue S, Yasui F, Yokochi S, Arai M, Morita K, et al. SARS-CoV spike protein-expressing recombinant vaccinia virus efficiently induces neutralizing antibodies in rabbits pre-immunized with vaccinia virus. Vaccine. 2007;25(4):630-7. Epub 2006/10/03. doi: 10.1016/j.vaccine.2006.08.039. PubMed PMID: 17011679; PubMed Central PMCID: PMCPMC7115632.

35. Tomori O, Kolawole MO. Ebola virus disease: current vaccine solutions. Curr Opin Immunol. 2021;71:27-33. Epub 2021/04/20. doi: 10.1016/j.coi.2021.03.008. PubMed PMID: 33873076.

36. Yoshikawa T, Taniguchi S, Kato H, Iwata-Yoshikawa N, Tani H, Kurosu T, et al. A highly attenuated vaccinia virus strain LC16m8-based vaccine for severe fever with thrombocytopenia syndrome. PLoS Pathog. 2021;17(2):e1008859. Epub 2021/02/04. doi: 10.1371/journal.ppat.1008859. PubMed PMID: 33534867; PubMed Central PMCID: PMCPMC7886154.

37. Watanabe S, Fukushi S, Harada T, Shimojima M, Yoshikawa T, Kurosu T, et al. Effective inactivation of Nipah virus in serum samples for safe processing in low-containment laboratories. Virology journal. 2020;17(1):151. Epub 2020/10/11. doi: 10.1186/s12985-020-01425-8. PubMed PMID: 33036623; PubMed Central PMCID:

PMCPMC7547523.

38. Omura N, Yoshikawa T, Fujii H, Shibamura M, Inagaki T, Kato H, et al. A Novel System for Constructing a Recombinant Highly-Attenuated Vaccinia Virus Strain (LC16m8) Expressing Foreign Genes and Its Application for the Generation of LC16m8-Based Vaccines against Herpes Simplex Virus 2. Jpn J Infect Dis. 2018;71(3):229-33. Epub 2018/05/02. doi: 10.7883/yoken.JJID.2017.458. PubMed PMID: 29709968.

39. Kaku Y, Noguchi A, Marsh GA, Barr JA, Okutani A, Hotta K, et al. Antigen capture ELISA system for henipaviruses using polyclonal antibodies obtained by DNA immunization. Arch Virol. 2012;157(8):1605-9. Epub 2012/05/16. doi: 10.1007/s00705-012-1338-3. PubMed PMID: 22585045.

40. Khetawat D, Broder CC. A functional henipavirus envelope glycoprotein pseudotyped lentivirus assay system. Virology journal. 2010;7:312. Epub 2010/11/16. doi: 10.1186/1743-422X-7-312. PubMed PMID: 21073718; PubMed Central PMCID: PMCPMC2994542.

41. Niwa H, Yamamura K, Miyazaki J. Efficient selection for high-expression transfectants with a novel eukaryotic vector. Gene. 1991;108(2):193-9. Epub 1991/12/15. doi: 10.1016/0378-1119(91)90434-d. PubMed PMID: 1660837.

42. Geisbert TW, Feldmann H, Broder CC. Animal challenge models of henipavirus infection and pathogenesis. Curr Top Microbiol Immunol. 2012;359:153-77. Epub 2012/04/06. doi: 10.1007/82_2012_208. PubMed PMID: 22476556; PubMed Central PMCID: PMCPMC7120132.

43. Kärber G. Beitrag zur kollektiven Behandlung pharmakologischer Reihenversuche. Naunyn-Schmiedebergs Archiv für experimentelle pathologie und pharmakologie. 1931;162(4):480-3.

44. Kaku Y, Noguchi A, Marsh GA, Barr JA, Okutani A, Hotta K, et al. Second generation of pseudotype-based serum neutralization assay for Nipah virus antibodies: sensitive and high-throughput analysis utilizing secreted alkaline phosphatase. Journal of virological methods. 2012;179(1):226-32. Epub 2011/11/26. doi: 10.1016/j.jviromet.2011.11.003. PubMed PMID: 22115786.

45. Brennan G, Stoian AMM, Yu H, Rahman MJ, Banerjee S, Stroup JN, et al. Molecular Mechanisms of Poxvirus Evolution. mBio. 2023;14(1):e0152622. Epub 2022/12/15. doi: 10.1128/mbio.01526-22. PubMed PMID: 36515529; PubMed Central PMCID: PMCPMC9973261.

46. Antoine G, Scheiflinger F, Dorner F, Falkner FG. The complete genomic sequence of the modified vaccinia Ankara strain: comparison with other orthopoxviruses. Virology. 1998;244(2):365-96. Epub 1998/05/28. doi: 10.1006/viro.1998.9123. PubMed PMID: 9601507.

47. Meyer H, Sutter G, Mayr A. Mapping of deletions in the genome of the highly attenuated vaccinia virus MVA and their influence on virulence. J Gen Virol. 1991;72 (Pt 5):1031-8. Epub 1991/05/01. doi: 10.1099/0022-1317-72-5-1031. PubMed PMID: 2033387.

48. Najera JL, Gomez CE, Domingo-Gil E, Gherardi MM, Esteban M. Cellular and biochemical differences between two attenuated poxvirus vaccine candidates (MVA and NYVAC) and role of the C7L gene. Journal of virology. 2006;80(12):6033-47. Epub 2006/05/30. doi: 10.1128/JVI.02108-05. PubMed PMID: 16731942; PubMed Central PMCID: PMCPMC1472566.

49. Yokote H, Shinmura Y, Kanehara T, Maruno S, Kuranaga M, Matsui H, et al. Vaccinia virus strain LC16m8 defective in the B5R gene keeps strong protection

comparable to its parental strain Lister in immunodeficient mice. Vaccine. 2015;33(45):6112-9. Epub 2015/08/05. doi: 10.1016/j.vaccine.2015.07.076. PubMed PMID: 26241947.

50. Kidokoro M, Tashiro M, Shida H. Genetically stable and fully effective smallpox vaccine strain constructed from highly attenuated vaccinia LC16m8. Proceedings of the National Academy of Sciences of the United States of America. 2005;102(11):4152-7. Epub 2005/03/09. doi: 10.1073/pnas.0406671102. PubMed PMID: 15753319; PubMed Central PMCID: PMCPMC554788.

Figure legends

Fig 1. Genome structure of the recombinant LC16m8 expressing NiV surface glycoproteins. Schematic diagrams of the procedure for constructing recombinant LC16m8 (upper) and genome structures of the foreign genes inserted into the LC16m8 genome (lower). Plasmid pRecB5R.1 expresses the mCherry and XGPRT genes under the vaccinia early and late promoter (P) and contains the homologous sequences that flank the B5R gene (UP and DOWN). The genes of interest (GOIs), which were cloned into the pRecB5R.1, were transferred to the LC16m8 genome through homologous recombination. The selection marker genes XGPRT and mCherry were self-excised from the intermediate recombinant viruses by intragenomic homologous recombination between the UP and shorter sequence homologous to UP (U’). The dotted lines indicate the expected locations of the homologous recombination sites. The final recombinant viruses express the NiV G or F gene, while the complementary genome DNA expresses the EGFP gene.

Fig 2. Expression and function of the envelope glycoproteins of NiV. (A and B) RK-

13 cells were infected with LC16m8-G or LC16m8-F at an MOI of 0.01. At 40 hpi, the cells were fixed and stained with a polyclonal antibody against NiV G protein (A) or F protein (B), followed by incubation with Alexa Fluor 488-conjugated anti-rabbit secondary antibody. The stained cells were observed under a phase-contrast or fluorescence microscope. (C) RK-13 cells were transfected with an expression plasmid driven by the vaccinia early and late promoter (pRecB5R.1-NiV-G, pRecB5R.1-NiV-F, or pRecB5R.1). At 24 h posttransfection, the transfectant cells were infected with LC16m8-G or LC16m8-F at an MOI of 0.01. At 24 hpi, formation of syncytia was observed under a fluorescence microscope.

Fig 3. Induction of neutralizing antibodies against NiV. (A) Experimental design and schedule of inoculations with LC16m8 and the recombinant LC16m8s. Seventeen hamsters were intramuscularly inoculated with 1×10^6 PFU of the LC16m8. At 4 weeks, three groups of animals consisting of 4-7 hamsters each were inoculated with 5×10^6 PFU of the LC16m8-G, 2.5×10^6 PFU each of LC16m8-G and LC16m8-F, or the growth medium (negative control) as a prime immunization. Half of each group (2-4 hamsters) were further inoculated with the same recombinant viruses as a boost immunization. Two weeks after the final dose of the recombinant LC16m8, all hamsters were euthanized, which was followed by serum collection. Neutralizing titers were analyzed using live NiV (B) or pseudo-typed VSV expressing secreted alkaline phosphatase (SEAP) bearing NiV G and F proteins (VSV-NiV-SEAP) (C). (D) Experimental design and schedule of inoculations with the recombinant LC16m8s. Single recombinant virus (LC16m8-G or LC16m8-F, 5×10^6 PFU/hamster) or mixture of the viruses (2.5×10^5 PFU each) was intramuscularly administered to hamsters once or twice at a 2-week interval without

preimmunization of LC16m8. Two weeks after the last inoculation with the recombinant virus, sera were collected from all hamsters. Neutralization titers were determined based on the assay using live NiV (E) or the VSV-NiV-SEAP (F). Bars show the mean values.

Fig 4. Effective induction of neutralizing antibodies by three immunization cycles with the recombinant LC16m8s in the VV-preimmunized hamsters. (A) Experimental design and schedule of inoculations with LC16m8 and the recombinant LC16m8s. Twenty-seven hamsters were intramuscularly inoculated with 1×10^6 PFU of the LC16m8. At 4 weeks, three groups of hamsters (n=8, 9 or 10) were inoculated with 2×10^6 PFU of the LC16m8-G (Group 1: VV-G-single-site), 2×10^6 PFU of the LC16m8-F (Group 2: VV-F-single-site) or both viruses (1×10^6 PFU each, onto two sites) (Group 3: VV-G-F-separate-sites). Two weeks after infection with the recombinant LC16m8s, one third of the hamsters in each group were inoculated once or twice at a 2-week interval with the same recombinant viruses as the prime inoculation. Two weeks after the last shot, serum samples were collected. Neutralizing titers were determined using live NiV (B). All groups of hamsters are shown with roman numerals in parentheses. Dotted line and bars show mean ± SEM. A one-tailed Student's t test assuming unequal variance was used for analyzing the data. ND_{50} values below the LOD (<2) were assigned a value of 1. Antibody titers were also determined using IFA method (C). BHK cells were transfected with the expression plasmid encoding NiV-G or NiV-F. At 24-36 hpi, the cells were fixed and reacted with dilutions of the hamster serum, followed by incubation with Alexa Fluor 488-conjugated anti-hamster secondary antibody. The stained cells were observed under a fluorescence microscope. IFA titers were defined based on the maximum serum dilution

in which a fluorescence signal was detected, and the values are shown as the average ± SEM.

Click here to access/download;Figure;LC16m8-NiV Figures 1234 Vaccine Watanabe et al.pdf

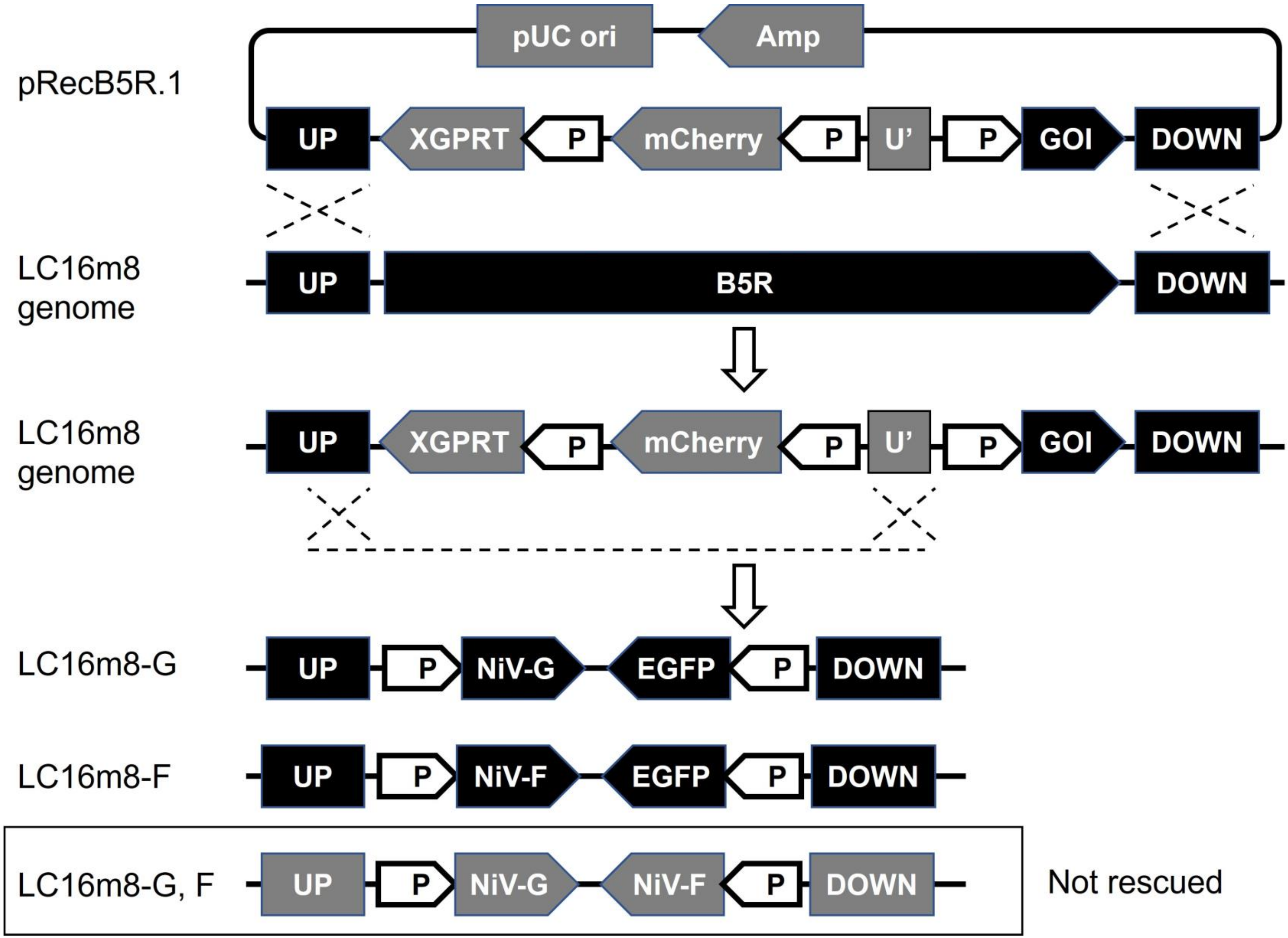

Figure 1, Watanabe et al

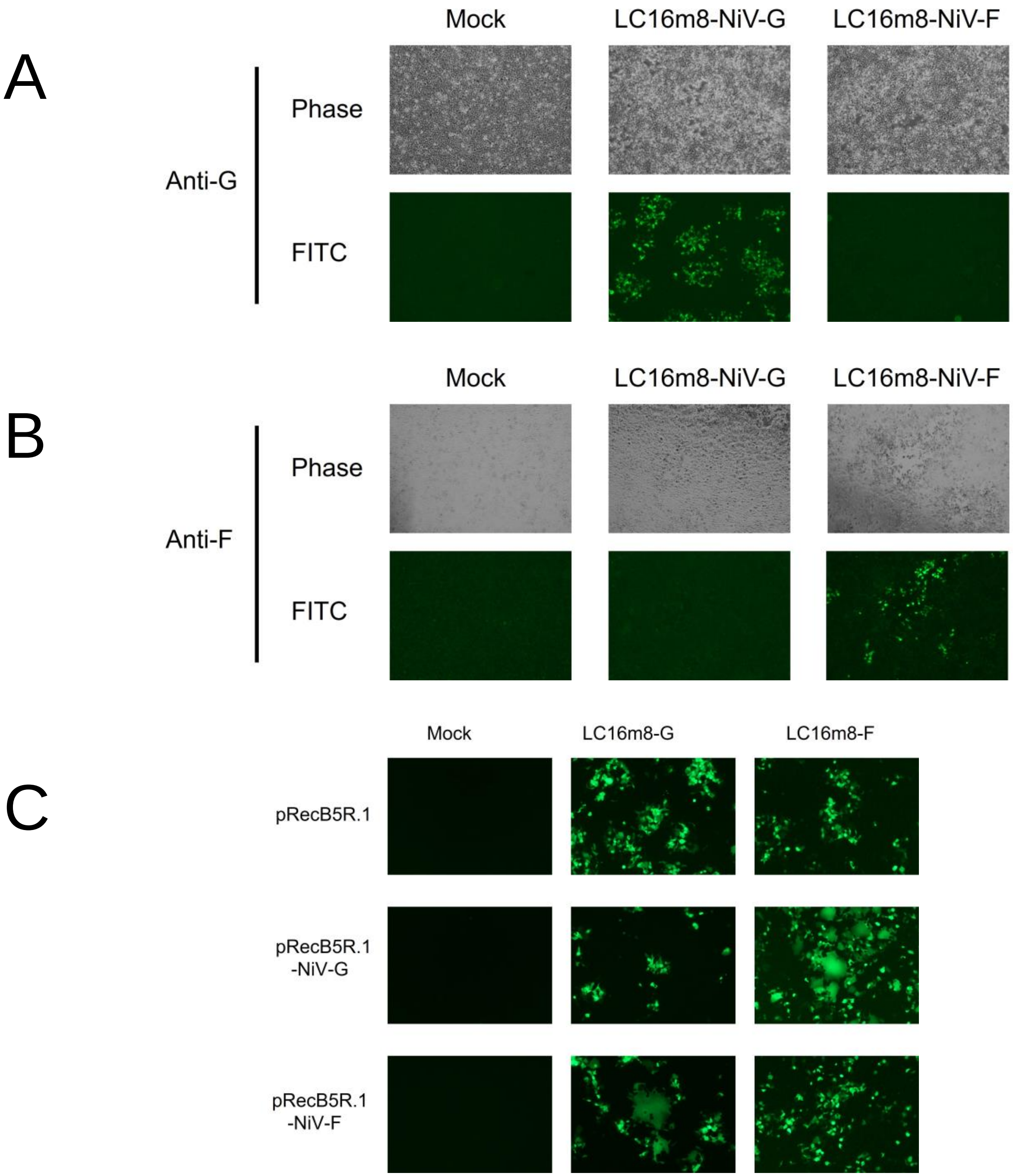

Figure 2, Watanabe et al

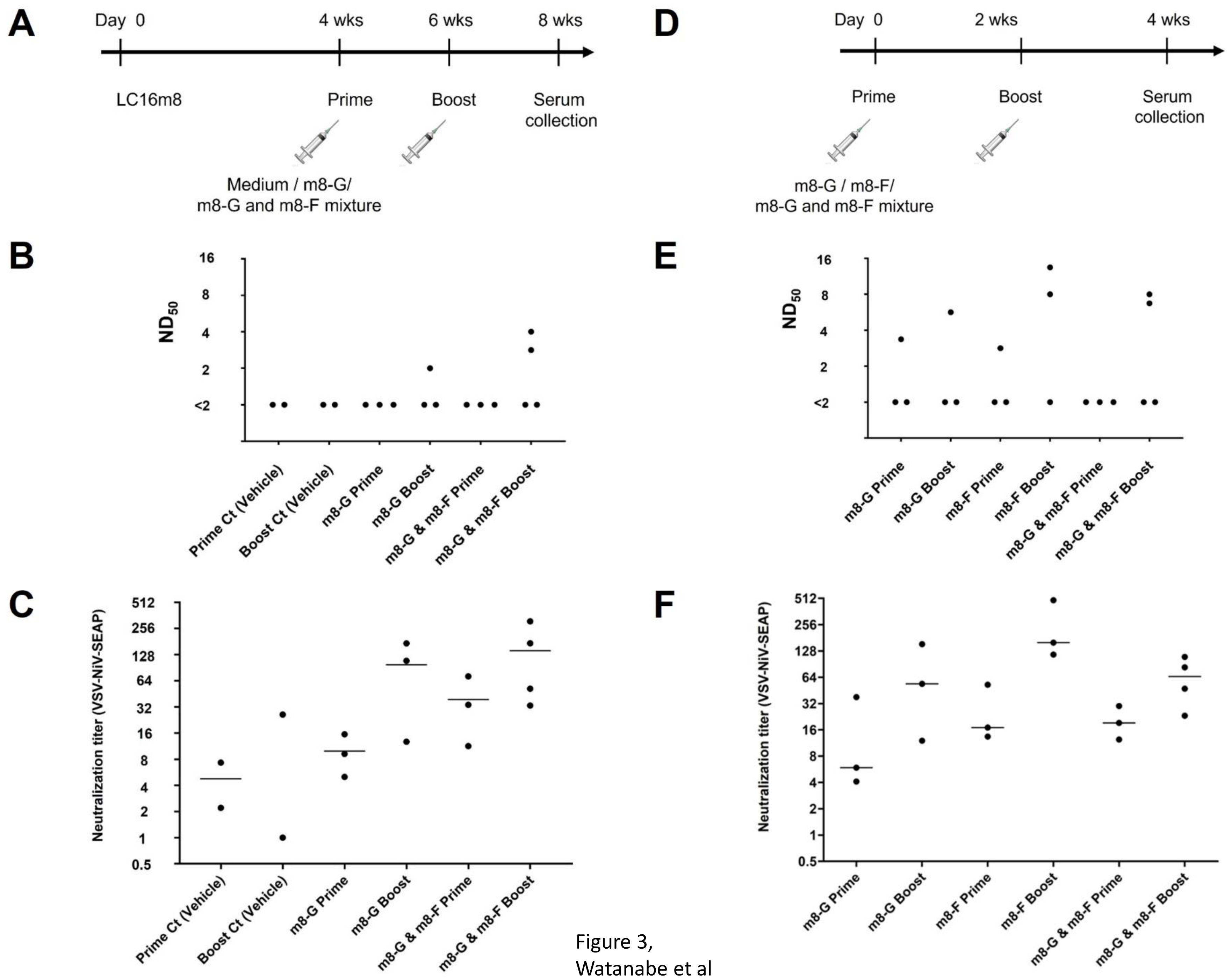

Figure 3,
Watanabe et al

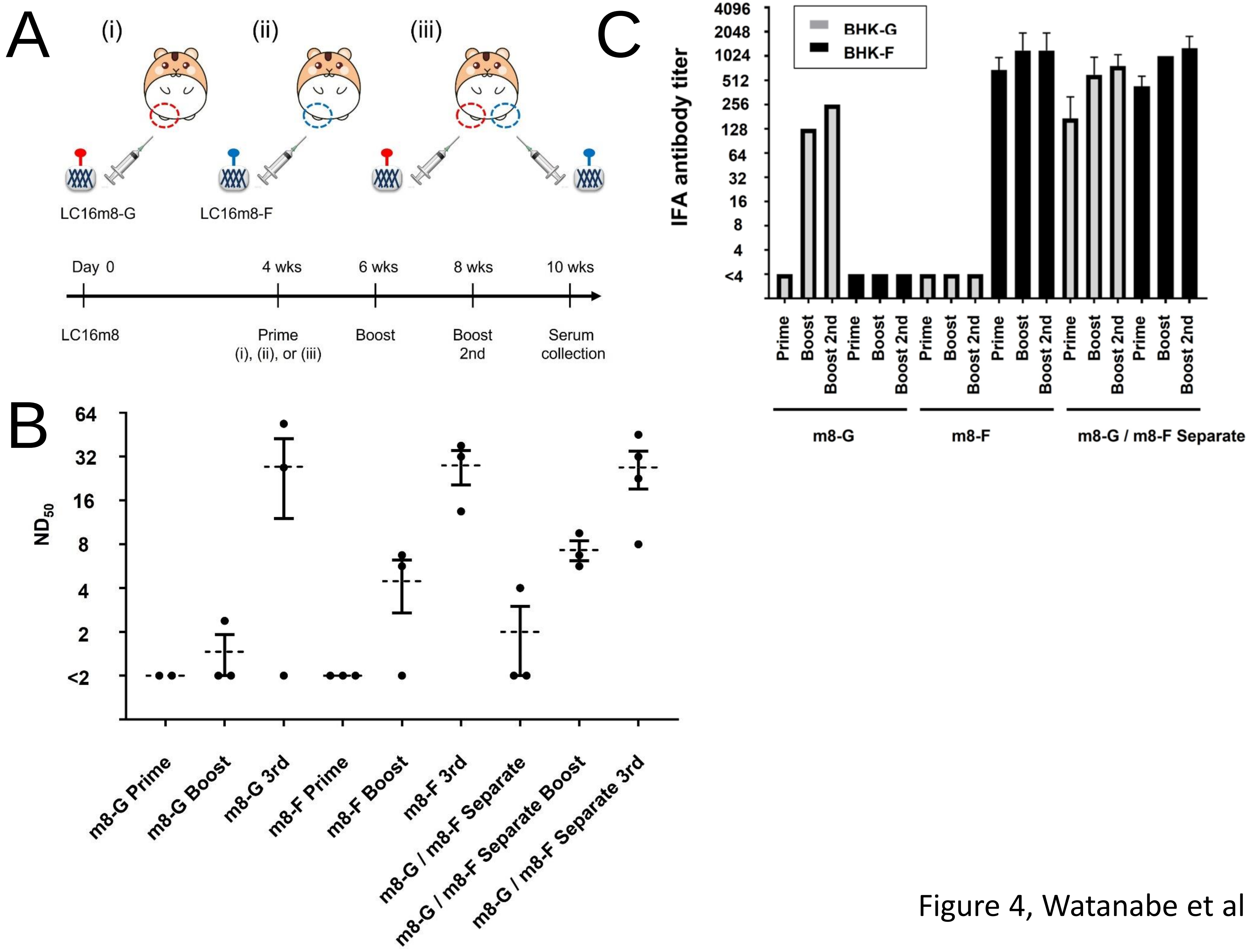

Figure 4, Watanabe et al

Click here to access/download
Supporting Information
Supporting Information.docx
