# Supplementary material for: Construction of a recombinant vaccine expressing Nipah virus glycoprotein using the replicative and highly attenuated vaccinia virus strain LC16m8
Source: PLoS Negl Trop Dis. 2023 Dec 15;17(12):e0011851. doi: 10.1371/journal.pntd.0011851 (PMC10756534; doi:10.1371/journal.pntd.0011851)
Supplement: Attachment — Submitted filename: Responses to the revieweres Watanabe_et_al_PLOS_NTD_230818.docx [file pntd.0011851.s002.docx]

We appreciate the kind and valuable suggestions and comments from both reviewers.

**Reviewer #1**

**<Methods>**

**Reviewer #1: Specific comments for the methods are detailed below**

**<Results>**

**Reviewer #1: Specific comments for the methods are detailed below**

**<Conclusions>**

**Reviewer #1: Specific comments for the methods are detailed below**

**< Editorial and Data Presentation Modifications?>**

The responses to the specific comments of Reviewer #1 in this section are as follows:

(**Line numbers with a shading background** in our response are those in the revised manuscript.)

**L9-14: Complete Affiliation of these footnotes should be mentioned - city, prefecture, etc.**

**>Response**

We have added complete information about the affiliations of the authors in **line 9-16**.

**L32: I suggest that full name will be included when mentioning the viral proteins for the first time in the text.**

**>Response**

According to the reviewer’s comment, the names of the proteins are described by their full names in **line 34**.

**L60-61: Any reference?**

**>Response**

We added a reference (**line 63)**.

**L89: I suggest adding some reference to the recent monkeypox outbreak that brought to attention the use of new VV-based vaccines, while addressing the undesired side effects.**

**>Response**

As suggested by the reviewer, we have added a sentence with a reference addressing safety properties (**line 96-99**).

**L123: I suggest adding "G or F GLYCOPROTEINS" or ANTIGENS for clarity**

**>Response**

To clarify antigens, the phrase was replaced by “ the NiV surface glycoproteins (G or F glycoproteins)” in **line 128-129**.

**L198: The number of animals in each group should be mentioned clearly to allow the reader evaluate the statistical significance of the results.**

**>Response**

Since the number of dots in Fig 3B, C, E, F, and Fig 4B corresponds to the number of animals, it may not be too difficult for the reader to evaluate the statistical information from the Figures. However, the number of animals is clearly described in the Methods section to help the reader understand, in accordance with the reviewer’s comment (**line 210-222, line 228-233)**.

**L207: What does this mean? They were vaccinated with VV earlier? Can the authors add some reference or refer the reader to a description of the background of the animals in this context?**

**>Response**

After pre-immunization with the original VV vaccine (LC16m8), the hamsters were immunized with LC16m8-based NiV vaccines. To clarify the meaning, the sentences have been modified, as in **line 210-216**.

**L274: I think that rephrasing to "****expression of mCherry under conditional drug selection", or something like that, and avoid using the word "influence" in that context.**

**>Response**

As suggested by the reviewer, the phrase was replaced by “ the expression of the mCherry gene under conditional drug selection with the MPA, xanthine, and hypoxanthine” (**line 279-281)**.

**L275: I did not understand what drives the order of recombinations? I understood that the selection of XGPRT drives the selection for the first recombination. But what drives the excision of the XGPRT-mCherry segment? Was the selection made by culturing only the GFP-positive plaques? Since this is a complicated procedure, I think that the authors may explain this , in order to make it easier for the reader to understand.**

**>Response**

As illustrated in Fig 1, the mCherry and XGPRT gene cassette is flanked by the sequences UP and U'. As already explained in the legend of Fig 1 (**line 757-763)**, the U' sequence is homologous to the sequence regions UP or DOWN but has a shorter sequence than both regions. Thus, as indicated by the dotted lines in Fig 1, in the absence of drug selection pressure, homologous recombination between UP and U' will occur and the gene cassette will be lost naturally (without any selection). Although the procedure is somewhat complicated, we have already reported the development of this method in our previous paper (this has also been mentioned in **line 276-278**). Therefore, we refrain from adding further explanations to the Result section. Instead, we have modified sentences in the Method section to help readers understand the procedure more easily (**line 184-188).**

**L276: I suggest that the authors will give the full name of the reagent, at least in the first time it is mentioned in the text.**

**>Response**

Although we cannot find any reagent name to be spelled out in L276, instead, we spelled out the “XGPRT” gene in the first place in the text (**line 183)**.

**L306: How many animals were in each group?**

**>Response**

To clarify the number of animals, we have added this information in the Results section (**line 314, line 335-337, and line 366)**, as well as in the Methods (**line 210-222, line 228-233)**.

**L311: this was a booster? if so, please mention it. if not, please explain the purpose of this step.**

**>Response**

We intend to explain the booster shots. To make this clear, we have modified the sentence in **line 318-319**.

**L315: I think that either "bearing...the...genes" or "expressing...the....proteins" should be used here.**

**>Response**

As suggested by the reviewer, the phrase was replaced by “ pseudovirus expressing the NiV G and F proteins” (**line 321-322)**.

**L332: the tests were PERFORMED not DETERMINED. the neutralization titer was determined.**

**>Response**

As already described in the original line 316, the titer of the neutralizing antibody was abbreviated as “SNT or pVSV-SNT.” Thus, we selected the word “determined.” To show this clearly, we have modified the sentence in **line 322-323**.

**L349: I think it should be Fig 4, not 3**

**>Response**

Based on the findings in Fig 3, we hypothesized that syncytium formation induced in cells infected with both LC16m8-G and LC16m8-F would inhibit the induction of antibodies, as described in **line 358-360**. Based on this hypothesis, we tested another inoculation method in hamsters (Fig 4A, iii). To explain this, we cited Fig 3B, C, E, and F (not Fig 4) in the sentence (**line 354-358).**

**L382: succeeded in generating,,,,, modified or recombinant virus**

**>Response**

Although we could not find the exact instructions or suggestions for this point, we have modified the sentence to help readers understand the text more easily (**line 391-392)**.

**L389: Figure 4C shows the average and SEM of each group, not individual animals. How can the reader understand that one animal in the G group did not produce anti-NiV G antibodies?**

**>Response**

In response to the reviewer's claim or indication, the original bar graph was replaced by the scatter plot (new “**Fig 4C**”).

**L408: If the authors performed physical inspection to identify any such reactions in the tested animals in this study, they should report this in the results. Otherwise, it should be stated that the animals were inspected regularly and did not show any clinical signs of adverse reaction to the inoculation.**

**>Response**

We observed the clinical signs of the hamsters daily. According to the reviewer’s comment, we have added an explanation in the Results (**line 346-347)** and Materials and Methods sections (**line 237-238).**

**L410: Was there any experiment that measured the viral load in the animals? either titration or quantitative PCR? If so, please refer the reader to the appropriate data. If not, this statement needs a different reference.**

**>Response**

We did not perform any experiments to measure viral load in hamsters. Since there is a correlation between the proliferative potential of VV strains in animals and the degree of skin lesions induced at the site of inoculation, it has been postulated that LC16m8 shows very limited proliferative potential in hamsters. To further emphasize the intention we would like to state in the discussion, we have added sentences with a few references (**line 433-437).**

**L428: Does this refer to human clinical trials? Is the LC16m8 used, or was it tested on humans? The authors should be very clear on the safety profile of this strain if the argue that it can be used as an alternative to other VV scaffolds for recombinant vaccines.**

**>Response**

As described in L107-109 (**line 111-114**), LC16m8 was approved in Japan as a licensed vaccine for smallpox, and its safety profile was evaluated by vaccinating more than 100,000 children, among whom serious adverse events were not reported. In Japan, 10,578 children participated in clinical trials of LC16m8 from 1973-1974 (Details are reported in two journals described in Japanese: **Rinshotovirus. 1975; 3:269-79**, **Shonikarinsho. 1976; 29:1409-12**). More than 3000 adults also participated in the clinical test in 2002-2010 in Japan [32, 33], whereas 125 adults participated in the USA [23] without serious adverse events. Based on its excellent track record of safety and efficacy in clinical trials as a smallpox vaccine, the World Health Organization recommends LC16m8 as a stockpiled vaccine for smallpox outbreak responses (<https://www.who.int/publications/i/item/WHO-WHE-IHM-2017-14>). Furthermore, as we have added a description in **line 96-99**, LC16m8 has also been the subject of attention as a monkeypox vaccine because it has the potential to protect against monkeypox infection and a good safety profile [26]. As described in **line 459-460**, clinical trials conducted in Japan have demonstrated that the safety of LC16m8 is maintained, even though reversion mutation could exist. Moreover, recombinant LC16m8 expressing exogenous viral proteins deletes the B5R gene region and replaces it with a foreign gene, thereby eliminating the possibility of reversion to the parental strain. To clarify this point, we have added a sentence in **line 460-462.**

**L433: a very low RATE - or - very low RATES**

**>Response**

As mentioned above, for LC16m8-G or LC16m8-F, reversion to the parental strain does not occur. Therefore, we are aware that it is inappropriate to describe this content in the context of a discussion. Therefore, we have deleted the sentence “**The occurrence of reversion at a very low rate may enhance the induction of humoral responses without enhancing virulence**.”

**L441-443: I did not understand whether the LC16m8 replicates in humans or not. If it does, than adverse side effects, secretion and reversion to virulence are a noticable concern. Recently, an attenuated VV strain was used for mokeypox vaccination, which does not replicate in humans, and it is therefore much safer for immunocompromised patients ( DOI: 10.1056/ nejmoa2215201). I think that the authors should address the option of using a single-cycle strain, such as the JINNEOS vaccine, for a safer vaccine scaffold.**

**>Response**

As described in **line 109-111** and **line 430-431**, LC16m8 has the potential to proliferate in humans while maintaining high attenuation in humans. That is why LC16m8 inoculation induces local skin reactions (small pock formation or “Take”) and erythema in humans [33]. As mentioned in response to the comment on **L428 of reviewer #1** (see above), clinical trials have demonstrated that the safety of LC16m8 is maintained, even with the possibility of a reversion mutation. Furthermore, LC16m8-G and LC16m8-F lack the B5R gene region; thus, reversion to the parental strain does not occur. Although we agree that MVA-based vaccines also show good safety profiles, it has recently been reported that two-shot MVA-BN immunization in humans yields relatively low levels of neutralizing antibodies against the monkeypox virus (Nat Med. 2023 Jan;29(1):270-278). In general, the ability of MVA-based vaccines to induce antibody responses is weak, probably because of their non-proliferative characteristics. As described in **line 467-473**, the LC16m8-based vaccine format has superior features as a proliferative vaccine compared to other poxvirus-based vaccines, including MVA-based vaccines.

**Figure Legends**

**L711 (Fig.1): Is there a particular reason that the selection and indicator genes were designed in an opposite orientation to the GOI? I am curious to know, as this might make the cloning less convenient, but there is no specific explanation for that.**

**>Response**

In DNA viruses with double-stranded DNA genomes, such as poxviruses and herpesviruses, when two viral genes are located in close proximity, the respective coding sequences of the two (or more) genes are not placed in series in the same direction on the same single-sided DNA strand but are often placed in opposite directions (one gene on one side of the DNA strand and the other on the complementary strand). This is possible because the viral proteins responsible for transcription and/or translation function in such a way that two independent events (i.e., the expression of two genes) do not interfere with each other when they act on transcription and translation. As illustrated in Fig 1, the NiV-G or EGFP gene is located on the viral genome to be expressed under the vaccinia early and late promoters (the poxvirus promoter sequence, "P") (**line 757-765**). Therefore, each pair of genes was designed to be efficiently expressed in opposite directions.

**L767: Average and SEM of how many repeats? Were technical repeats for each serum sample? or one well/plate for each sample? Please provide details on the experiment and number of repeats.**

**>Response**

Two weeks after the last injection with recombinant LC16m8s, serum samples were collected from each animal. Subsequently, the neutralizing or IFA titers were determined using a single test. To clarify this, we have added sentences in **line 805-808**. As mentioned above (in response to the comment on **L389 of reviewer #1**), Fig 4C has been replaced with a scatter plot to show each titer clearly. Accompanying this replacement, we have added a description of the graphic display for the dot plot, as in **line 816-818**.

<Summary and General Comments>

Responses to the Summary and General Comments by reviewer #1 are as follows.

**The study described in this manuscript is definitely important and address an important need to help the efforts to overcome and prevent Nipah virus infections. There are some issues, however, that I think need to be addressed by the authors to render the manuscript ready for publication in PNTD.**

**The authors claim that the constructs they produced are superior to those developed by Guillaume et al. in 2003 (doi: 535 10.1128/jvi.78.2.834-840.2004.). while in the 2003 study, the authors demonstrate actual protection in a challenge experiments, both after immunization and as passive immune response (antibody administration), in the present manuscript, no such challenge experiment is described. The authors indeed show that immunization with both G- and F-expressing recombinant viruses induces significant neutralizing antibodies, but in the absence of a challenge experiment, their argument, in my opinion, is not firm. In order to support the major claim of this study, i.e., convince that the LC16m8 NiV vaccine is a promising alternative, I suggest that the authors will include a more detailed comparison of their results to those of Guillaume et al., for the parts that were performed in both studies. This way, the reader can appreciate the outcomes of the two studies in a comparative manner.**

**>Response**

Although we agree that further challenge studies with NiV are required to confirm the efficacy of recombinant LC16m8s as a NiV vaccine, as already described in **line 428-429**, we believe that our findings and comparison with a prior report suggest that the LC16m8-based vaccine is more effective than the NYVAC-based vaccine, especially in inducing neutralizing antibodies. To explain this, we have added sentences in **line 399-418** in the Discussion section, in accordance with the reviewer’s suggestion.

**Also, I suggest that the authors will revise the relevant parts of the discussion section, to incorporate a part that addresses the limitations of the present study, including the number of animals, the absence of a comprehensive safety study, and the absence of a challenge experiment.**

**It is somewhat difficult to argue that the new vaccine scaffold is superior without a challenge experiment to demonstrate it, so a more detailed comparison of the existing data might strengthen it nonetheless.**

**>Response**

In response to the specific comments by reviewer #1 (see above), we have added information on the number of animals (**line 210-222, line 228-233), line 314, line 335-337, and line 366**) and replaced the original Fig 4C with a scatter plot to clarify the number of animals. Secondly, as mentioned above in **the response to L428** (see above), the safety of LC16m8 has been demonstrated in clinical trials. Concerns regarding reversion mutations are not relevant to recombinant LC16m8s, because their B5R regions are replaced with a foreign gene (NiV G or F gene). These results support the safety of LC16m8-G and LC16m8-F. Third, we have added a description that the LC16m8-based vaccine is more effective than the NYVAC-based vaccine at inducing neutralizing antibodies as mentioned above (**line 399-417**). We believe that all modifications in the text strongly support the idea that the LC16m8-based vaccine format has superior characteristics as a proliferative vaccine.

**Another issue is the complete dataset. The authors show in some cases average values, and I think that a supplementary table or tables with the values obtained for each animal, depending on the experiment, will be helpful for the reader to appreciate the work. Additionally, this is a requirement of the journal.**

**>Response**

As described in the response to **the specific comment to L767,** Fig 4C has been replaced by a scatter plot.

**As I mention below in the specific comments regarding the discussion section, I suggest that the authors will address the option of using a non-replicative VV scaffold, such as the one used for the recent JYNNEOS vaccine, as a safer option. Since it cannot replicate in the human body, it has significantly fewer and less severe side effects and contraindications, compared with previous VV-based vaccines, some of which are mentioned in this manuscript.**

**>Response**

As described in the response to **the specific comment to L441-443,** the safety of LC16m8 was demonstrated and established. Although we agree that MVA-based vaccines also show good safety profiles, their ability to induce antibody responses is not necessarily strong, as mentioned above. LC16m8 is a promising viral vector vaccine that can induce a stronger humoral immune response based on its relatively high or modest proliferative potential while maintaining high attenuation in humans.

**Lastly, although the language is good overall, I noticed some sentences that need to be rephrased or corrected. It is likely that I missed some others, so a second language check might be useful before submitting a revised version.**

**In conclusion, I think that the manuscript could be suitable for publication in PNTD, after the authors will address the points raised in this review.**

**>Response**

Following the reviewer's suggestion, we have used an English editing service. We appreciate the valuable comments from Reviewer #1.

**Reviewer #2**

**<Methods>**

**Objectives are clearly stated with a clear testable hypothesis stated. The study design appropriately addresses the stated objectives. Hamsters are well-documented animal models for human Henipavirus infection and are appropriate for the hypothesis being tested. Three (3) groups of hamsters (Group G-single-site, F-single-site, and G/F-single-site) were inoculated to evaluate antibody induction by the recombinant viruses (LC16m8-G, LC16m8-F). Similarly, three (3) groups of hamsters (Group VV-G-single-site, VV-G/F214 single-site, and VV-mock-single-site) harboring pre-existing immunity against VV were inoculated. However, the authors did not indicate the number of Hamsters used for each group. Authors could state, for example, that 2 hamsters each were inoculated for each group. This should be stated in the Animal Experiment section, and not just the figure legends. Statement in Lines 206-210 under the subheading 'Animal Experiment' is repeated in Lines 210-214. Appropriate statistical analysis was used, and there were no ethical concerns.**

**>Response**

As described above, in response to the comments on **L198 and L306 of reviewer #1**, we have added information on animals in the Methods section (**line 210-222, line 228-233**), as well as in the Results section (**line 314, line 335-337, and line 366**). Accompanying these modifications, we have deleted the repeated statement pointed out by Reviewer #2.

**<Results>**

**Analysis presented match the analysis plan, and results are clearly and completely presented.**

**<Conclusions>**

**Conclusions are supported by the data presented with clearly stated limitations. The successful induction of neutralising antibodies against Nipah virus (NiV) in hamsters using LC16m8-based vaccine format provides an important basis for the clinical use of all vaccinia virus-based vaccines against NiV disease. This is well articulated by the authors.**

**<Summary and General Comments>**

**The study looks good and original with public health relevance. Laboratory experiments were carefully carried out and in detail. The manuscript was well written.**

**>Response**

We appreciate reviewer #2 for his favorable and valuable comments.
